# Building a benchmark dataset for the Kurdish news question answering

**DOI:** 10.1016/j.dib.2024.110916

**Published:** 2024-09-06

**Authors:** Ari M. Saeed

**Affiliations:** Computer Science Department, College of Science, University of Halabja, Kurdistan Region, Halabja, Iraq

**Keywords:** Kurdish question answering system, Kurdish news dataset, Data mining, Text pre-processing, Machine learning

## Abstract

This article presents the Kurdish News Question Answering Dataset (KNQAD). The texts are collected from various Kurdish news websites. The ParsHub software is used to extract data from different fields of news, such as social news, religion, sports, science, and economy. The dataset consists of 15,002 news paragraphs with question-answer pairs. For each news paragraph, one or more question-answer pairs are manually created based on the content of the paragraphs. The dataset is pre-processed by cleaning and normalizing the data. During the cleaning process, special characters and stop words are removed, and stemming is used as a normalization step. The distribution of each question type is presented in the KNQAD. Moreover, the complexity of the QA problem is analyzed in the KNQAD by using lexical similarity techniques between questions and answers.

Specifications Table

**Table:** 

Subject	Applied Machine Learning
Specific subject area	Text Classification, Question Answering System, Data Mining
Data format	Raw Cleaned Normalized Stemming Table
Type of data	Text
Data collection	The ParsHub software is used for extracting and collecting data from Kurdish news websites. The question-answering pairs are made manually based on the content of each paragraph.
Data source location	Kurdish News Websites
Data accessibility	Repository name: (KNQAD): Kurdish News Question Answering Dataset [1]. Data identification number: DOI: 10.17632/tc28knsfsn.1 Direct URL to data: https://dx.doi.org/10.17632/tc28knsfsn.1

## 1 Value of the Data

• *Resource for Low-Resource Language***:** This is the first attempt to create a question-answering dataset for a low-resource language such as Kurdish. It is useful for automatically extracting answers from questions and responding to frequently asked questions. In addition, it can be used to evaluate the accuracy of AI-generated tools and then compare it with human answers.
• *Domain-Specific Data***:** Focuses on news content, offering domain-specific data that can enhance the accuracy and relevance of question-answering systems within the news context.
• *Support for Kurdish NLP Models***:** Enables the development and fine-tuning of Kurdish language models, contributing to better language understanding, text analysis, and information retrieval in Kurdish.
• *Benchmark for Future Research***:** Serves as a benchmark dataset for evaluating and comparing the performance of different QA models in the Kurdish language, fostering further research and innovation.
• *Tool for Multilingual NLP***:** Contributes to the growing collection of multilingual QA datasets, helping to develop more robust and inclusive NLP models that support multiple languages, such as Arabic, Persian, and Urdu, since the dataset uses Arabic script, which is written from right to left.
• *Cultural and Linguistic Preservation***:** Supports the preservation and promotion of the Kurdish language in digital spaces, contributing to its use and recognition in modern technology.
• *Educational and Research Resource***:** Provides a valuable resource for educators, students, and researchers interested in Kurdish language processing, machine learning, and AI. Moreover, the dataset is valuable for analyzing and evaluating various models by splitting the data for training and testing. Furthermore, it is a good resource for assessing algorithms in chatbot systems.

## 2 Background

Creating datasets is one of the most important challenges in Natural Language Processing (NLP). It is important to note, that for accurately detecting and classifying subjects in NLP, the first step is preparing a suitable dataset. The Kurdish language is one of the least resourced languages in all fields, especially in the field of NLP, compared with languages such as English, French, and Chinese. The main objective of this experiment is to create the Kurdish News Question Answering Dataset (KNQAD) as a first attempt for researchers and students working on automatic question-answering systems, machine learning, data mining, and deep learning. The second objective is pre-processing the data to be ready for implementing various models. The raw texts are extracted from multiple news websites, and the dataset is collected and pre-processed to be prepared for implementing different models.

## 3 Data Description

In this dataset, the Kurdish language is used to collect the text. Sorani and Kurmanji are two widely spoken dialects of the Kurdish language. The scripts and text direction of each dialect are different. In Sorani, the text is written in Arabic from right to left, while Kurmanji uses Latin script from left to right [2,3]. In this dataset, the Sorani dialect is used to collect the texts. The alphabet of the Sorani dialect is almost similar to the Arabic alphabet. The number of letters in the Sorani alphabet is 36 divided into vowels and consonants, while in Arabic, there are 28 letters [4], as shown in Table 1.

**Table 1:** Kurdish and Arabic alphabets.

As shown in Table 1, two of the Kurdish letters  use the same script and the same phonetic sound for each vowel and consonant, but they differ based on their position in words, as shown below:

The  in the words , meaning (Kurd, smart), are vowels, while the  in the words , meaning (destroyed, memories), are consonants. The Sorani dialect uses the same numbering and punctuation scripts as the Arabic language  [5].

**Fig. 1:**
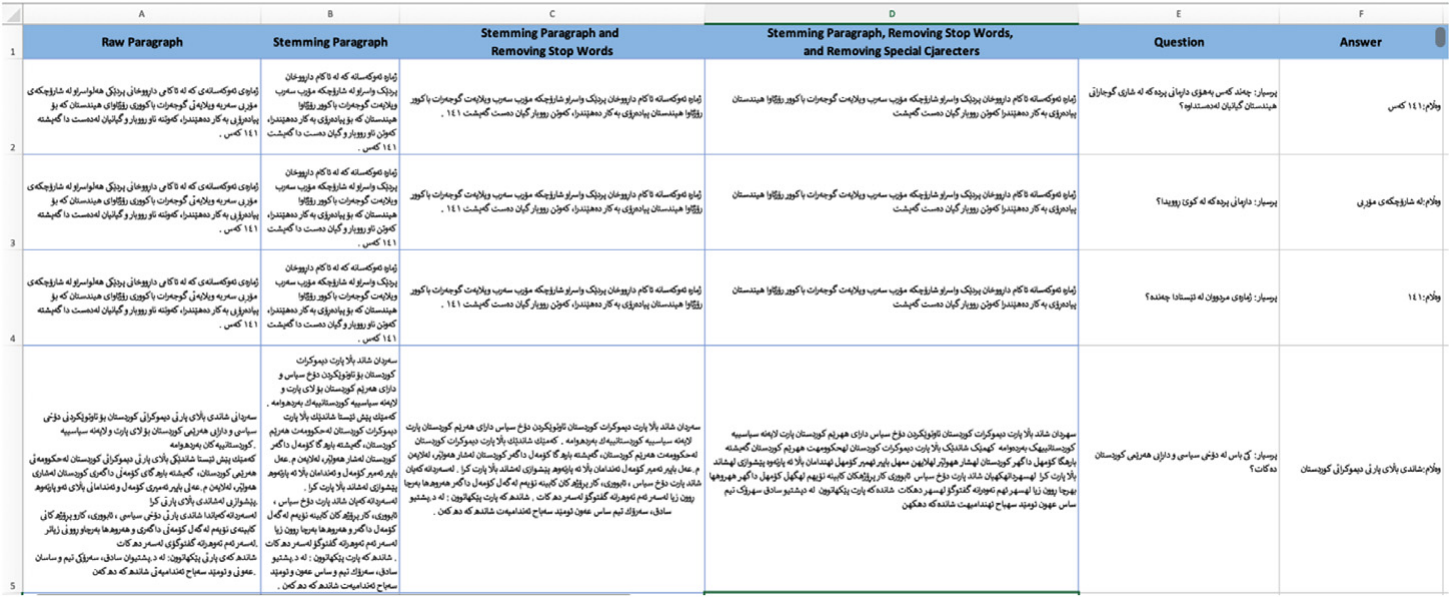
Contents of the Kurdish news question answering dataset. as shown in Fig. 1, each column contains specific text data as defined below:.

The texts of the dataset are saved in Mendeley data as (KNQAD) Kurdish News Question answering Dataset.xlsx with 6.6 MB. The Excel file consists of 15,003 rows and six columns. The first row is the header row (name of the column) while the rest of the rows are text data as shown in Fig. 1:

The first column is the (Raw Paragraph) without any preprocessing.

The second column is the (Stemming Paragraph).

The third column is the (Stemming Paragraph and Removing Stop Words).

The fourth column is the (Stemming Paragraph, Removing Stop Words, and Removing Special Characters).

The fifth column is the (Questions)

The six-column is the (Answers)

To annotate KNQAD, three annotators manually annotated the dataset. All annotators have a linguistic background and they are native Kurdish speakers. The author of this study acted as a super-annotator while the rest of them have experience as annotators. The following steps are implemented to create the question and extract the answer:

• The first step involves one annotator reading the news paragraph.
• The second step requires the other two annotators to understand the paragraph.
• In the third step, these two annotators create questions based on the content of each paragraph.
• In the fourth step, the initial reader (first annotator) answers the questions based on the content of the paragraphs.

Determining the relevance of a paragraph to a given question is crucial in building effective question-answering (QA) systems. Keyword matching is used to double-check the answers within the paragraphs, while synonyms and paraphrases are used to create the questions. Moreover, annotators in this dataset followed a set of guidelines and procedures as part of the annotation protocol to ensure the data's quality, accuracy, and consistency [6]. This protocol typically includes detailed instructions on how to annotate various components of the dataset, such as questions, answers, and context paragraphs. Below are the key components considered in KNQAD:

As shown in Table 2, the annotators follow the annotation protocol to create a benchmark question-answering dataset. In addition, the questions are designed to cover a wide range of topics, balancing straightforward factual information with more complex reasoning tasks. The difficulty varies to assess different levels of understanding, and a mix of question types ensures comprehensive model training. Each question is crafted to be relevant to the paragraph, clear, and applicable to real-world scenarios. These considerations contribute to creating a versatile and effective QA dataset. Moreover, in KNQAD, the types of questions vary based on the content of each paragraph. Knowing the question types and their occurrences helps us understand how to work with the dataset and how the models predict appropriate answers [7]. In addition, there are eight types of questions used in KNQAD, as shown in Table 3:

**Table 2:** Question-answering annotation protocols.

Annotation protocols	Detailed overview of key components
1. Objective and Scope	**Objective:** The objective of this experiment is to create a benchmark dataset by providing a clear paragraph for the annotation. **Scope:** The questions were written based on the content of the paragraph, and the answers are selected and included in the paragraph.
2. Selection of Context	**Guidelines for Context Selection:** Relevant paragraph have been selected and documented that contain the information needed to answer the questions. **Relevance Criteria:** Keyword matching is used for selecting the answers, while topical relevance and proximity of information are used as criteria for determining the relevance of questions in a paragraph.
3. Question Annotation	**Question Types:** Different types of questions, such as fact-based, opinion-based, and yes/no questions, are created and handled. **Clarifications:** Ambiguous or unclear questions are addressed and reviewed.
4. Answer Annotation	**Answer Extraction:** Instructions on how to select or generate the correct answer from the paragraph are provided for span-based QA. This includes marking the exact start and end positions of the answer. **Acceptable Variations:** Annotators are guided to accept keyword matches to answer the question in the paragraph, while synonyms, paraphrases, or alternative correct answers are not acceptable. **Handling Unanswerable Questions:** There are no 'unanswerable' labels, as all answers are included in the paragraph.
5. Quality Control	**Review Process:** Peer review and expert review are conducted by annotators, while an automated review is performed using simple Python code to check for matching keyword answers within the passage. **Conflict Resolution:** In cases of conflicts or disagreements between annotators, issues are escalated to a super-annotator or resolved in consensus meetings.
6. Ethical Considerations	**Bias Mitigation:** Annotators are provided with instructions to avoid cultural and linguistic biases when annotating the dataset. **Sensitive Content:** Harmful and sensitive text is removed from the content.
7. Documentation and Reporting	**Reporting Anomalies:** Unusual or problematic cases that may require changes or removal from the dataset are reported.
8. Tools and Software:	**Annotation Tools:** The annotation process was done manually; therefore, no platform was used. **File Formats:** The dataset is saved in Excel file (.xlsx extension).
9. Final Validation	**Quality Assurance Checks:** Final checks are conducted on the annotated dataset to ensure that all guidelines have been followed and that the data meets the required standards. **Feedback Loop:** A feedback loop is established where annotators can report issues with the protocol and suggest improvements.

**Table 3:** Question types of Kurdish language.

**Fig. 2:**
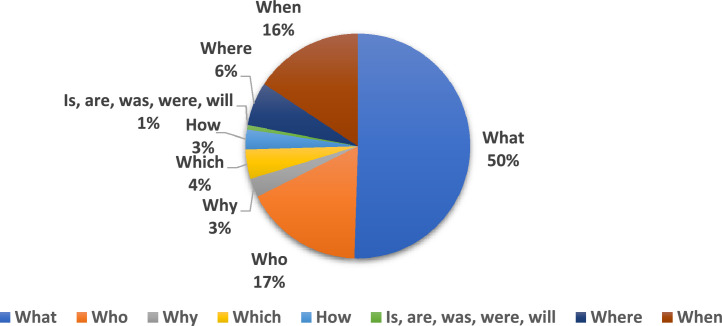
Question types distribution over KNQAD.

Table 3 shows examples of question types in the Kurdish language, with their translations into English. Another important point is analyzing the distribution of the question types in KNQAD, as shown in Fig. 2.

As shown in Fig. 2, ‘whatʼ is the most frequent question type, while ‘is, are, was, were, willʼ is the least frequent question type. Moreover, it is crucial to highlight the complexity of lexical similarity between questions and answers in KNQAD. As the questions become more complex, finding the answers becomes increasingly difficult [8]. Table 4 shows the effectiveness of the similarity measure between questions and answers for two question-answer pairs.

**Table 4:** The similarity between question and answer.

Table 4 shows the similarity between two questions and one answer. The Jaccard Coefficient equation is used to decide which question is close to being selected as a main question for the answer by using the similarity measure between the questions and the answer [9]. The Jaccard Coefficient compares each word in the answer with each word in the questions; if the word occurs in both the question and the answer, it counts as 1, and if it does not, it counts as 0. After comparing all the words, the Jaccard Coefficient equation divides the number of common words by the total number of words in the answer plus the total number of words in the questions, as shown in the following equation:

(1) $$\text{Jaccard}\;\text{Coefficient}\;\left(\text{Question},\;\text{Answer}\right)=\frac{No.\;common\;words\;in\;question\;and\;answer}{No.\;word\;in\;Question+No.\;words\;in\;Answer}$$

$$\text{Jaccard}\;\text{Coefficient}\;\left(\text{Question}\;1,\;\text{Answer}\right)=\frac{2}{6+8}=0.14$$

$$\text{Jaccard}\;\text{Coefficient}\;\left(\text{Question}\;2,\;\text{Answer}\right)=\frac{5}{8+8}=0.31$$

Based on Eq. (1), the answer is related to the second question since the similarity between question 2 and the answer is 0.31 and is greater than the similarity between question 1 and the answer, which is 0.14.

## 4 Experimental Design, Materials, and Methods

One of the most important challenges in natural language processing is predicting a good model by implementing various algorithms on a suitable dataset. For collecting text data and creating a dataset for some languages such as English, Arabic, and Chinese numerous methods and techniques are considered due to a large volume of resources in contrast with the Kurdish language which is the low resource.

In this experiment, to collect an appropriate dataset, more than twelve Kurdish News websites are selected to extract the data. The URLs are chosen in various fields such as social news, religion, sport, science, and economy. The amount of data is different for each URL as shown in Table 5:

**Table 5:** Websites name with amount of data.

No	Name of Websites (URL)	Amount of Data
1.	https://kurdistantv.net	13 %
2.	https://politicpress.com	8 %
3.	https://hengaw.net	9 %
4.	https://rudaw24.net	4 %
5.	https://www.rudaw.net/sorani	10 %
6.	https://www.xendan.org	5 %
7.	https://www.kurdsatnews.com	11 %
8.	https://www.peyam.net	9 %
9.	https://www.speda.net	7 %
10.	https://www.kurdistan24.net	14 %
11.	https://channel8.com	4 %
12.	https://www.knnc.net	6 %

As shown in Table 5, various websites are selected with different ratios to collect the text. For extracting the text from the above websites, the ParsHub tool is selected as an extracting tool for scraping the text from the URL. In Parshub, starting and extracting the texts from the URL, five steps should be followed as shown below:

The first step is creating an account by using an email and password.

The second step is creating a new project.

The third step is to enter a URL to extract the data.

**Fig. 3:**
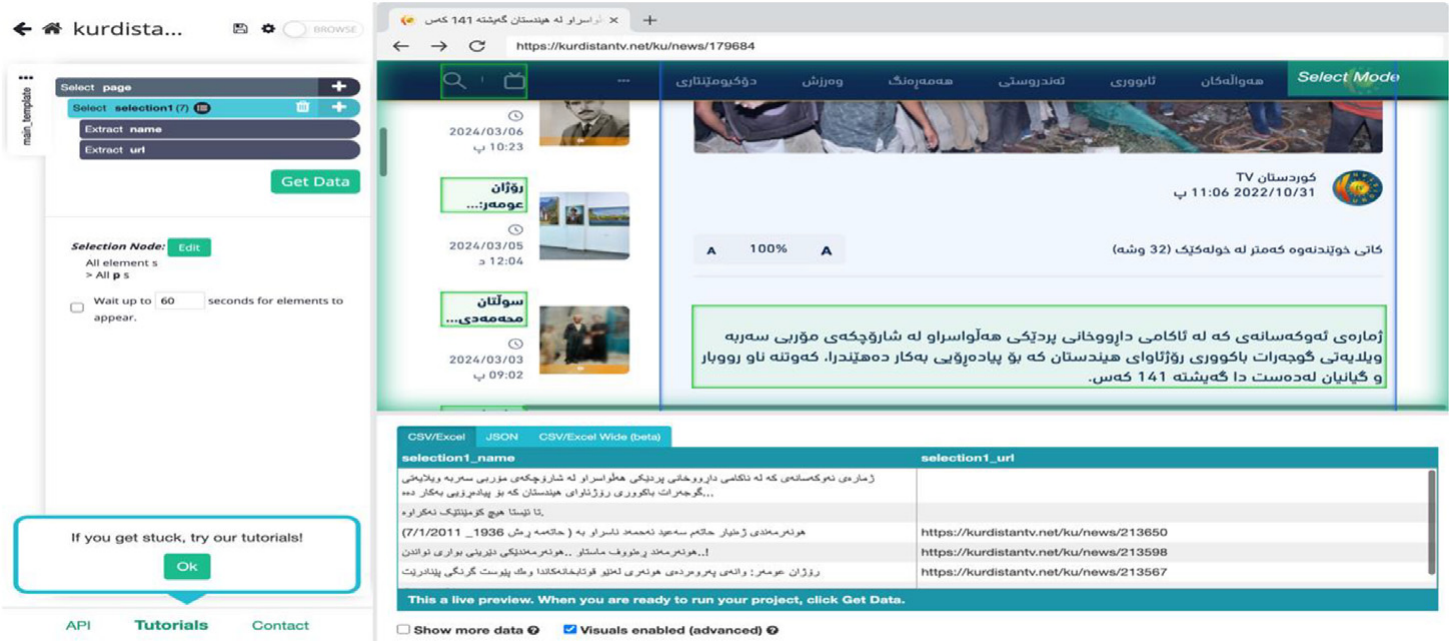
Data extraction from ParsHub.

The fourth step is selecting the number of pages as shown in Fig. 3.

As shown in Fig. 3, the ParsHub interface software consists of two parts. The left-hand side provides a text field for entering the URL and controlling the number of pages while the right-hand side is the result of the URL which is a website from the top and extracting the texts from the bottom.

**Fig. 4:**
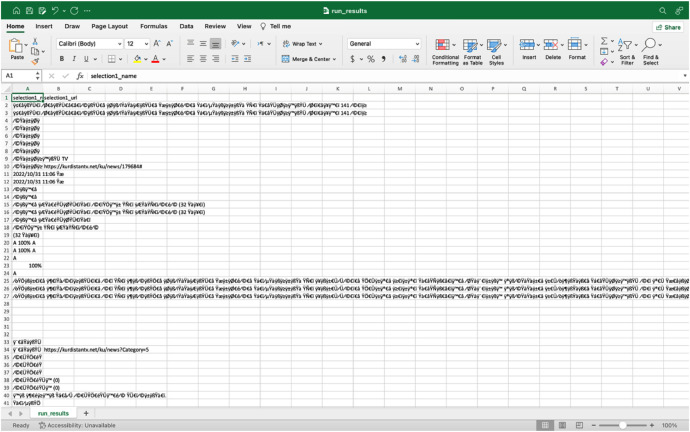
CSV result set.

The fifth and final step is getting the data and extracting it as a CSV file as shown in Fig. 4.

**Fig. 5:**
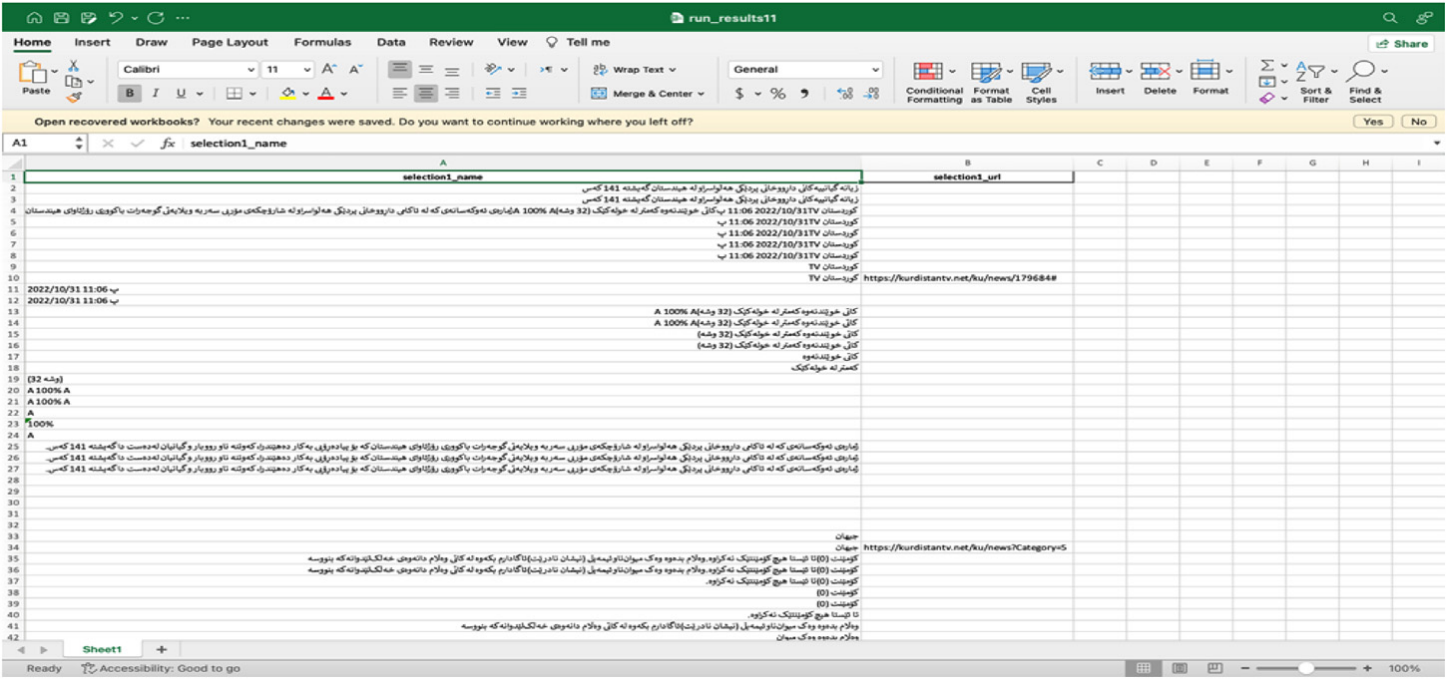
Read Kurdish font in Xlsx file.

As shown in Fig. 4, the CSV result set includes Kurdish text that has been transformed into a special font, making it unreadable. To fix this issue, the Python code is used to read the CSV files and rewrite them in XLSX files as shown in Fig. 5.

As illustrated in Fig. 5, the special code is converted into a readable Kurdish font, which can be used to gather the dataset. After collecting the dataset, another step is text preprocessing.

**Fig. 6:**
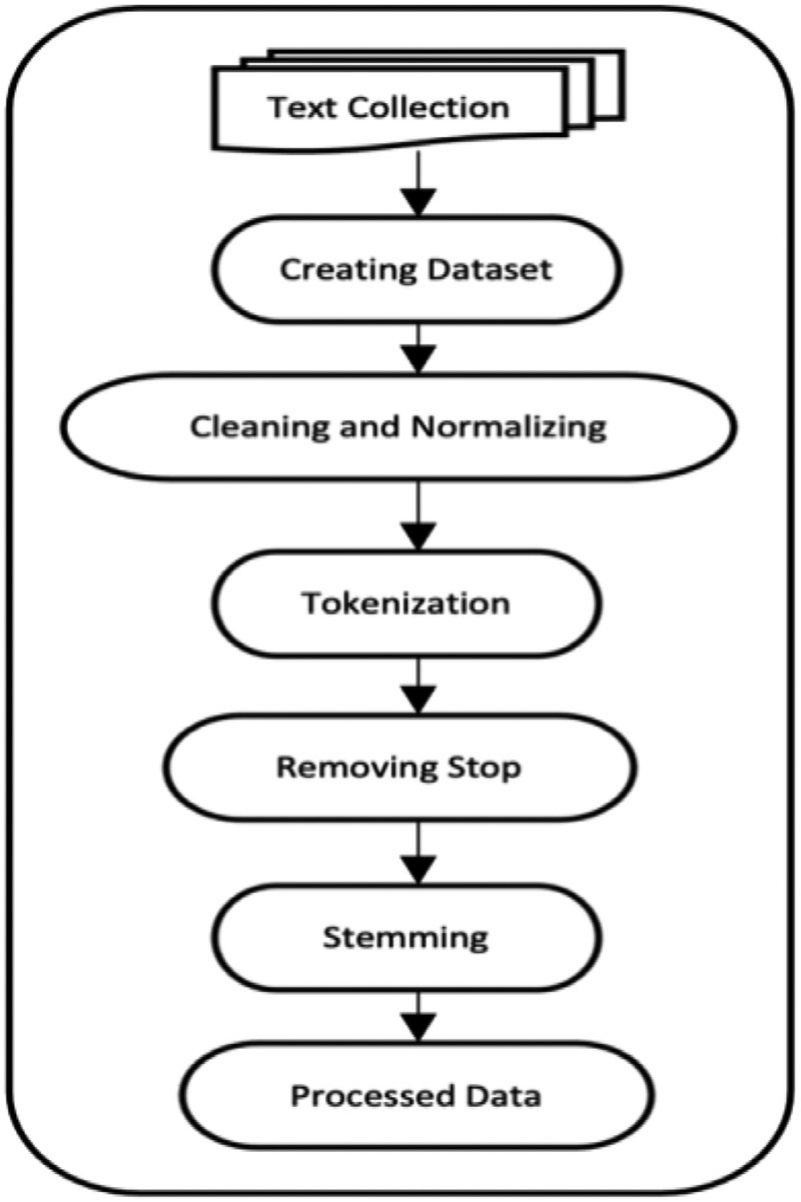
Text pre-processed.

Text preprocessing in NLP involves converting raw text into a clean and standardized format, making it suitable for using machine learning models. The main aim of preprocessing techniques for any language is to remove some special characters, decrease the high dimensional of the text, and keep the relevance of the text. Removing nonrelevant words and special characters increases models' accuracy and time consumption [10]. Moreover, the preprocessing steps are different from one language to another language based on the morphology and the syntax of the languages. The morphology of the Kurdish Language is complex since it needs an adequate step for preprocessing the texts for that purpose [11,12], the following preprocessing steps are implemented on the dataset by using the Python code as shown in Fig. 6:

As shown in Fig. 6, the text is preprocessed after collecting and implementing steps as explained below:

• The First step is text collection: the text is extracted and collected from different websites.
• The Second step is creating the dataset: The dataset is created from collection data.
• The Third step is cleaning and normalizing the dataset: the dataset is cleaned by removing some special characters, numbers, and punctuation such as () and the dataset is normalized with reforming the different script: replace , replace, replace, replace, replace , , replace, and replace since different types of keyboards are used for writing the texts.
• The Fourth step is Tokenization: tokenization is the process of splitting sentences into words. In the Kurdish language, spaces are used to separate words within sentences.
• The fifth step is removing stop words: stop words are a set of words in any language which is repeated in texts and do not have a special meaning. In this experiment, 240 stop words are removed.
• The sixth step is stemming, which involves removing prefixes and suffixes from words to find their root form. To achieve this, 25 rules based on word length are applied to the dataset [2].

Table 6 shows the preprocessing steps after implementing the KNQAD:

**Table 6:** Pre-processing KNQAD.

As shown in Table 6, the text is preprocessed by removing special characters, eliminating stop words, and stemming.

## Limitations

The process of data collection and implementing pre-processing steps needs much time since the personnel computer is used for all experiments due to the lack of a supercomputer.

## Ethics Statement

The author confirms their adherence to the ethical requirements for publication in Data in Brief. The research work conducted does not involve human subjects, animal experiments, or the use of data collected from social media platforms. It is important to note that the text of this dataset is collected from a news website which is already shared and applicable to the data collection process. Moreover, the dataset does not contain any personal information and does not violate any organization and parties. The texts are standardized according to legal and ethical guidelines and policies.

## CRediT authorship contribution statement

**Ari M. Saeed:** Supervision, Data curation, Conceptualization, Methodology, Visualization, Funding acquisition, Writing – original draft, Writing – review & editing, Software.

## Data Availability

(KNQAD): Kurdish News Question answering Dataset (Original data) (Mendeley Data).
